# Determinants of the continuous operations of micro and small enterprises during COVID-19 pandemic in Ethiopia

**DOI:** 10.1186/s13731-021-00187-z

**Published:** 2021-11-01

**Authors:** Erstu Tarko Kassa

**Affiliations:** Woldia University, Woldia, Ethiopia

**Keywords:** Micro and small enterprises, Continuity, COVID-19, Partnership, Leadership

## Abstract

The main objective of this study is to assess determinant factors for the continuous operations of micro and small enterprises during COVID-19 pandemic. The study adopted a cross-sectional  design, with both descriptive and explanatory research design. To achieve the objectives of the study, 276 respondents were selected from 890 micro and small enterprise owners. The sample of the study was selected through proportional stratified random sampling technique from the business types (manufacturing, construction, urban agriculture, service and trade). To collect the primary data, questionnaires were dispatched to owners/operators of micro and small enterprises. The collected data were analyzed through descriptive, correlation and regression analysis techniques. The finding of the study revealed that people and administrative factors, regulatory factors, economic factors, partnerships, leadership of owner have a positive relationship to micro–small enterprise continuous operations during COVID-19 pandemic with the value of *r* = 0.457, 0.558, 0.572, 0.519 and 0.654, respectively. The study regression analysis result assured that partnership, economic factors, and leadership of the owner has a positive statistical significant effect on the continuous operations of the micro and small enterprise during COVID-19 pandemic with the value of (*p* < 0.05). The researcher recommended that strenghtening partnership with stakeloders and excersing best leadership practices are essential to ensure the continuous operations of the micro and small enterpreses.

## Introduction

Micro and small enterprise (hereafter MSE) has great contributions for the community, government and graduates or job seekers by creating job opportunities and enhance revenue for the individuals (Rascón & Velázquez, 2019). MSEs are contributing a lion share of the country economy to bring sustainable development in developed and developing countries, and able to bring a paradigm shift for a new and more sustainable way of production and consumption (Alemu & Dame, 2017; Karanja et al., 2013; Ruchkina et al., 2017).

The recent studies results show that micro and small enterprises contribute 60% of GDP and over 70% of total job opportunities in developing countries, and also above 95% of employment and 70% of GDP arise from MSE in the middle-income level countries (Abdissa & Fitwi, 2016). Even though, the contribution of MSEs become higher and significant in both developing and developed countries these enterprises have been ached by different disasters in different countries. Their continuity became in question by natural disasters and other catastrophic events (Samantha, 2018).

After the outbreak of COVID-19, the continuity of MSEs in different countries is not a smart way. The pandemic smashes the country's economy and has an impact on the output, disruption of supply chain, inflation, decline transportation services, investment declined, jobless citizens increased, and social impacts like poverty gender inequality. The pandemic forced the countries to closes non-essential businesses, bring bankruptcy for business, and laid off permanent and blue collar workers (Elenev et al., 2020; Techno Serve Business Solution to Poverty, 2020).

The COVID-19 pandemic has an impact on the MSEs, a response measure should be taken by the concerned bodies. It has an indirect impact on the Ethiopian MSE in the current situation due to the slow spread of the virus. The UNDP-supported Entrepreneurship Development Centre (EDC) has conducted a quick survey to assess the impact MSEs have sustained as a result of COVID-19, their perception of potential future impacts, and their recommendations for remedial actions. The data gathered from 60 EDC clients in Addis Ababa and in the four regional states where EDC has regional coordination offices (Amhara, Oromia, SNNPS, and Tigray) show that, on average, an enterprise lost Birr 142,654 (USD 4755) in monthly sales (80%) and Birr 21,643 (USD 721) in monthly income. Based on these figures, EDC clients alone, totaling 32,250 enterprises, have lost Birr 4.6 billion in monthly sales and Birr 704 million in monthly income during March 2020 (ILO, 2020; UN, 2020).

Previous studies indicate that the continuity of MSEs affected by financial sources, taxation, administration barriers, age, gender, scholarship, marital status, economic sectors, family tradition, increase income, profession, experience, flexible schedule, unemployment, financial support, problem, problems related with the sale, workers, wage finance, knowledge empowerment, capacity building, and location of the business (Alvarado Lagunas. et al., 2018; Ruchkina et al., 2017; Uchehara, 2019).

Based on the above discussion, the researcher was motivated to know which factors considerably affected MSEs’ continuity in this critical time of the COVID-19 outbreak. Among the factors, people and administrative factors, regulatory factors, economic factors, partnerships, the leadership of owner, and government support are examined under this study.

By considering the factors two objectives have been set by the researcher. The first objective is to evaluate to what extent people and administrative factors, regulatory factors, economic factors, partnerships, and leadership of owner affect the continuity of MSEs during COVID-19 and the second one is to examine the relationship between people and administrative factors, regulatory factors, economic factors, partnerships, and leadership of owner with MSE continuity during COVID-19 pandemic.

## Review of related literatures

Sustainability of MSE is the way to nonstop able learning, adapt and develop, renew, reconstruct, and reorient to maintain a lasting and distinctive position in the market by offering buyers above-average value today and in the future through organic variation constituting business models, and arising from the creation of new opportunities, objectives, and responses to them, while balancing the interests of different groups. Sustainable business development involves the application of sustainability principles to business operations. It is a variety of things like ecological sustainability, social sustainability, or sustained economic growth (Grudzewski et al, 2010; Szczepańska-Woszczyna & Kurowska-Pysz, 2016).

### MSEs’ continuity strategy

According to empirical studies when micro and small enterprises are unable to prepare a continuity plan, especially during disaster or crisis 75% of the business will fail due to natural and manmade disasters (Cook, 2015). During the crisis time, effort should be expended to prepare a contingency and a business plan that may help to mitigate, and restore micro and small businesses caused by the crisis (Cook, 2015; Quarantelli et al., 2007).

There is also a strategy to survive the micro–small enterprises, namely marketing innovation through fast distribution, proper promotion, and fair prices (Fabeil et al., 2020). Many studies on crisis management that help to save MSEs include at least three standard phases, i.e., pre-crisis, during crisis, and post-crisis (Fabeil, et al., 2019; Leinonen, 2018), which are usually further divided into more detailed phases. These may include risk assessment, prevention, preparedness, response, recovery, and learning, which are particularly used in the field of disaster reduction and business continuity as suggested by the notion of ISO standard (Leinonen, 2018). This notion is used in analyzing the results of the current study to understand the impact of the crisis on business strategy throughout each phase of movement control order (MCO) amid COVID-19 in Malaysia.

MSEs and industry relationships between the estimated risk of contagion at work and the adoption of robots, to test the hypothesis that robotization may facilitate social distancing and lower the risk of contagion. The analysis, which includes various controls of possible automation-related confounding factors and addresses possible issues of indignity, provides evidence that industries employing more robots per worker in production tend to exhibit a lower risk of contagion due to COVID-19 (Piccolo et al., 2020).

The other study done in Mexico, micro-business owners continue operating their micro-business with a strong relationship with family traditions, entrepreneurship benefits, and a low paying job. The variables identified in this study considerably affect the success of micro and small enterprises (Alvarado Lagunas et al., 2018).

Properly administering and managing the newly established business is crucial to survive and save the life of the business and has a positive effect. On the other hand, lack of study regarding pandemic outbreak that affects the enterprises negatively and the age of business, record and borrowing were seen as significant in predicting business success (Alemu & Dame, 2017; Fabeil et al., 2020).

In a study conducted by Kamunyu and Theuri (2017), they identified factors that affect the performance of women micro and small enterprises. Among the factors that affect the business, performance is inadequate capital, lack of business skills, and lack of access to credit facilities. A survey result shows that during COVID-19 pandemic 40% could not set a timeframe to continue due to wages and rents cost and 64% of MSE faced for bankruptcies by a labor shortage and supply chains and consumer demand.

According to Bouey (2020), physical space and unable to getting shop is a challenge for supermarkets, traditional food markets, restaurants, car dealers, movie theaters, gyms, and bars, suffered significant losses. The corona pandemic forced governments to lockdown was very severe in Italy, with a reduction in the value of potential output produced peaking at 69% for the construction and real estate and 63% for mechanics. As a result, GDP is expected to drop by around 10% in 2020 (Navaretti et al., 2020).

A study was done in China, South Korea, Japan, Italy, the UK, and the four largest states in the US to collect data related with work and living situations, income, behavior, beliefs about the COVID-19 pandemic, and exposure to the virus, socio-demographic characteristics and pre-pandemic health characteristics. The data indicate that calibrating certain parameters used in economic and epidemiological models, or for documenting the impact of the crisis on individuals, both in financial and psychological terms and for understanding the scope for policy intervention by documenting how people have adjusted their behavior as a result of the COVID-19 pandemic and their perceptions regarding the measures implemented in their countries (Michèle et al., 2020). Increased business start-ups, use of indigenous technologies, business, and customer growths help to enhance and improved to achieve sustainable rural development by operating MSEs (Uchehara, 2019).

### Conceptual framework of the study

See Fig. 1.

**Fig. 1:**
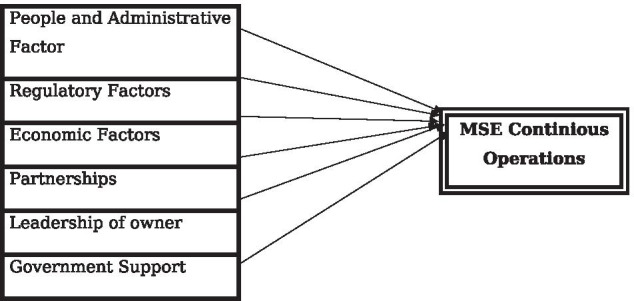
Conceptual framework of the study. *Source* Model proposed by the researcher (2020)

## Methods of the research

In this study cross-sectional method with both descriptive and explanatory research design has been used. Because the data were collected at one shot of time and described as it is by using descriptive research design and explanatory research design helps to explain the cause-and-effect relationship between independent variables with the dependent variable**.**

The target population of the study was 890 enterprise owners in the Woldia city administration. From the total population 214 owners engaged in the manufacturing sector, 96 construction, 75 urban agriculture, 238 services, and the remaining 267 were working in the trade sector (WTVEDO, 2020).

To participate in respondents from this study, the researcher used simple random sampling techniques from the targeted population of the study. This sampling technique allows for all target population equal chance to participate in this study. The sample size also has been determined by using Yamane (1967) formula and proportional technique from each business type. Thus, the formula is described as follows:

$$n = \frac{N}{{1 + N(e)^{2} }},$$

where *N* = target population, *n* = sample size, *e* = error term.

$$n = \frac{890}{{1 + 890\left( {0.05} \right)^{2} }}$$

$$n=276.$$

Based on the sample size the respondents have participated proportionally as follows from each business type. As shown in Table 1, the total population was divided as strata based on the business type owners engaged in.

**Table 1:** Sample size of the study

Business type	Number of MSE	Sample size
Manufacturing	214	66
Construction	96	30
Urban agriculture	75	23
Service	238	74
Trade	267	83
Total	890	276

*Source* TVEDO (2020)

### Data collection and measures

The data were collected by using a questionnaire technique from the respondents. All questionnaires contain a Likert scale. The source and the items are described as following in Table 2**.**

**Table 2:** Questionnaire items, measures and sources

Construct	Number of items	Measurement	Source
PAF	PAF1, PAF2, PAF3, PAF4, PAF5, PAF6 and PAF7	Likert scale (5 points)	ILO (2020)
Partnerships	PA1,PA2,PA3,PA4,PA5,PA6 and PA7	Likert scale (5 points)	ILO (2020)
Economic factors	EF1,EF2,EF3,EF4,EF5,EF6, and EF7	Likert scale (5 points)	ILO (2020)
Regulatory factors	RF1,RF2,RF3, and RF4	Likert scale (5 points)	ILO (2020)
Leadership of the owner	LO1,LO2,LO3, and LO4	Likert scale (5 points)	Szczepańska-Woszczyna (2016)
Government support	GS1,GS2,GS3,GS4,GS5, and GS6	Likert scale (5 points)	Tadesse (2020)
Continuous operation of micro and small enterprises during COVID-19 pandemic	MSEC1, MSEC2, MSEC3, and MSEC4	Likert scale (5 points)	Tadesse (2020)

### Model specification

As indicated by Kothari (2004), standard multiple linear regressions being one family of statistical techniques, is used to explore the relationship between one dependent variable and a number of independent variables or predictors simultaneously enabling to get answers regarding how well a set of variables is able to predict a particular outcome and which variable in a set of variables is the best predictor of an outcome. Thus, this study allowed multiple regression analysis used to understand by how much each independent variable (*people and administrative factors, regulatory factors, economic factors, partnerships, leadership of owner, and government support*) explains the dependent variable (continuous operations of micro and small enterprises during COVID-19 pandemic).

Regression equation of performance on selected variables:

$$Y \, = \, \beta_{0} + \, \beta_{1} X_{1} + \, \beta_{2} X_{2} + \, \beta_{3} X_{3} + \, \beta_{4} X_{4} + \, \beta_{5} X_{5} + \, \beta_{6} X_{6} + \, e,$$

where *Y* is a dependent variable—MSEs’ continuity during COVID-19 pandemic; X_1_ = *people and administrative factors*, X_2_ = *regulatory factors*, X_3_ = *economic factors*, X_4_ = *partnerships*, X_5_ = *leadership of owner*, and X_6_ = *government support*, are the explanatory variables. Β0 is the intercept term—constant which is equal to the mean if all slope coefficients are β_0_. β_1_, β_2_, β_3_, β_4_, β_5_, and β_6_, are the coefficients associated with each independent *t* variable which measures the change in the mean value of Y, per unit change in their respective independent variables and e is an error term.

## Results and discussion

### Demographic variables analysis

Table 3 indicates that 36.6% of respondents are female and the remaining 63.4% are male. From the total respondents, 7.6% were below the age of 20, 66.7% are between 21 to 30 years, 24.3% are between 31 and 40 years and the remaining 1.4% are above 40 years. Regarding business type respondents engaged in almost 23.9% are engaged in the manfacturing sector, 10.9% of respondents have participated in the construction business sector, 8.3% from urban agriculture, 26.8% are service business owners, and 30.01% are engaged in the trade sector.

**Table 3:** Demographic variables and business type

Variables	Frequency	Percent
Sex		
Male	175	63.4
Female	101	36.6
Total	276	100.0
Age		
< 20 years	21	7.6
21–30 years	184	66.7
31–40 years	67	24.3
> 40 years	4	1.4
Total	276	100.0
Types business engaged in		
Manufacturing	66	23.9
Construction	30	10.9
Urban agriculture	23	8.3
Service	74	26.8
Trade	83	30.1
Total	276	100

*Source* Own survey (2020)

### Descriptive statistics of independent and dependent variables

As indicated in Table 4, the mean result of the variables of the study for people and administrative factors, partnerships, economic factors, regulatory factors, leadership of owner, and government support has a value of 3.59, 3.36,2.89,3.01, 3.33, and 3.72, respectively. As per the cutoff point set by Al-Sayaad et al. (2006), the mean result for people and administrative factor and government support is indicated “Agree” for Likert scale level, the remaining variables indicated “neutral” level. Thus, the study done by Rascón and Velázquez (2019) the mean result of people and administrative factors is not similar to this study result.

**Table 4:** Descriptive statistics

Variables	Mean	Std. deviation	Response decision: Al-Sayaad et al. (2006)
People and administrative factors	3.5963	0.69579	Agree
Partnership	3.3639	0.82890	Neutral
Economic factors	2.8980	1.01719	Neutral
Regulatory factors	3.0100	1.11238	Neutral
Leadership of owner	3.3342	0.99677	Neutral
Government support	3.7271	0.75141	Agree
MSE continuous operations	3.4239	0.82026	Agree

*Source* Own survey (2020)

### Reliability statistics

To check the consistency and stability of the collected data, the reliability test is crucial. In this regard, the reliability test was conducted for six independent variables and one dependent variable of the study. The Cronbach’s alpha as shown in Table 5 for people and administrative factors, partnership, economic factor, regulatory factor, the leadership of owner, government support, and MSE continuity are 0.826, 0.833, 0.861, 0.860, 0.877, 0.795 and 0.757, respectively. As stated by Zikmund (2011), scales with a constant between 0.80 and 0.95 are considered to have very good reliability. Scales with a constant between 0.70 and 0.80 are considered to have good reliability, and a value between 0.60 and 0.70 indicates fair reliability. Therefore, the study variables reliability is above 0.7, which is a good reliability result as stated by Zikmund (Table 6).

**Table 5:** Reliability

Variables	Cronbach's Alpha	No. of items
People and administrative factors (PA)	0.826	7
Partnership (PR)	0.833	7
Economic factor (EF)	0.861	7
Regulatory factor (RF)	0.860	4
Leadership of owner (LO)	0.877	4
Government support (GS)	0.795	6
MSE continuous operations MSCO)	0.757	4

*Source* Own survey (2020)

**Table 6:** Correlations

	PA	PR	EF	RF	LO	GS	MSCO
PA	1	0.594**	0.556**	0.440**	0.513**	0.024	0.457**
PR	0.594**	1	0.561**	0.508**	0.600**	0.025	0.558**
EF	0.556**	0.561**	1	0.735**	0.659**	0.068	0.572**
RF	0.440**	0.508**	0.735**	1	0.679**	0.026	0.519**
LO	0.513**	0.600**	0.659**	0.679**	1	0.041	0.654**
GS	0.024	0.025	0.068	0.026	0.041	1	− 0.027
MSCO	0.457**	0.558**	0.572**	0.519**	0.654**	− 0.027	1
	0.000	0.000	0.000	0.000	0.000	0.653	

**. Correlation is significant at the 0.01 level (2-tailed). Source Own survey (2020)

### Correlation analysis

We can understand that except the government support other five independent variables has a positive relationship with micro and small enterprise continuity during COVID-19 pandemic. The variables ''r'' value of people and administrative factors, regulatory factors, economic factors, partnerships, leadership of owner which has a positive relationship with the value of *r* = 0.457, 0.558, 0.572, 0.519, and 0.654, respectively, with a *p*-value of less than 0.05.

Table 7 presents how much of the change in the explained variable is explained by the model. The multiple coefficients of determination denoted as *R*^2^ is 0.493. The value of the adjusted *R*^2^ indicates that 48.2% of the variance in the dependent variable was explained by the model.

**Table 7:** Model summary

Model	*R*	*R*^2^	Adjusted* R*^2^	Std. error of the estimate
1	0.702^a^	0.493	0.482	0.59026

^a^Predictors: (constant), GS, PA, RF, PR, LO, EF

When we come to the variables incorporated under in this study partnership, economic factors, and leadership of owner significantly (*p* < 0.05) influence positively the continuous operations of the MSEs COVID-19 pandemic, as indicated in Table 8. However, the remaining people and administration factors, regulatory factors, and government support do not significantly (*p* > 0.05) influence positively the continuous operation of MSEs during the COVID-19 pandemic.

**Table 8:** Coefficient

Model	Unstandardized coefficients		Standardized coefficients	*T*	Sig
	B	Std. error	Beta		
(Con)	1.350	0.261		5.180	0.000
PAF	0.041	0.068	0.035	0.611	0.542
PA	0.193	0.060	0.195	3.229	0.001
EF	0.148	0.058	0.184	2.565	0.011
RF	− 0.002	0.051	− 0.002	− 0.036	0.971
LO	0.331	0.055	0.402	6.032	0.000
Government support	− 0.068	0.048	− 0.062	− 1.426	0.155

*Source* Own survey (2020)

## Discussion

From the study variable government support has a negative relationship with MSE continuous operation during COVID-19 pandemic with the value  of − 0.027 with a *p* value of greater than 0.05. The correlation between people and administrative factors is similar to the finding of Abdissa and Fitwi (2016) with the result of *r* = 0.342. The other study was done by Rascón and Velázquez (2019) people and administrative factors had a positive relationship with other study variables with the value of *r* = 0.60 with significant *p* value. This study result is similar to this study. Another study was done by Cherkos et al. (2018) legal and leadership with MSE performance result in a high correlation with Pearson correlation values of *r* = 0.988 and 0.939, respectively. This indicates that there is a similar result to this study's findings.

The effect analysis finding revealed that leadership of the owner has positive influence on MSEs’ continuous operation during COVID-19 pandemic. This result is similar to the finding of Szczepańska-Woszczyna (2014). The role of leadership skills is crucial to sustain and enhance the performance of MSE in challenging times (Alemayehu & Gecho, 2016; Gelgelu, 2018; Kamunyu & Theuri, 2017).

In another study done by Tekola and Gidey (2019), weak human resources development schemes, dependency on government are the factors that influence the sustainability of Ethiopian micro and small enterprises. This result contradicts the present study’s findings because the people and administration factors and government supports are not significant under this study. Economic factors have a positive influence on MSEs’ continuous operation during COVID-19 pandemic.

## Conclusion

This study intended to investigate the factors determine the MSEs’ continuity during COVID-19 pandemic in Woldia City administration. From the factors incorporated under this study, people and administrative factors, regulatory factors, economic factors, partnerships, and leadership of owner have a positive relationship with the continuity of MSEs’ continuity during COVID-19 pandemic with the Pearson correlation value of *r* = 0.457, 0.558, 0.572, 0.519 and 0.654, respectively, with *p* value of less than 0.05. And also from the identified variables partnership, economic factors, leadership of owners have a positive influence on the continuity of MSE during COVID-19 pandemic with a *p* value of less than 0.05. The researcher recommended that strenghtening partnership with stakeloders and excersining best leadership practices are essential to ensure the continuous operations of the micro and small enterpreses.

## Data Availability

All data are included in the manuscript and available on hand too.

## Abbreviations

MSE: Micro and small enterprises
PA: People and administrative factors
PR: Partnership
EF: Economic factor
RF: Regulatory factor
LO: Leadership of owner
GS: Government support
MSCO: Micro and small enterprises continuous operations
